# Bronchopulmonary dysplasia: a predictive scoring system for very low birth weight infants. A diagnostic accuracy study with prospective data collection

**DOI:** 10.1007/s00431-021-04045-8

**Published:** 2021-04-06

**Authors:** Ikbel El Faleh, Mohamed Faouzi, Mark Adams, Roland Gerull, Jamel Chnayna, Eric Giannoni, Matthias Roth-Kleiner, for the Swiss Neonatal Network

**Affiliations:** 1 Clinic of Neonatology, Department Women-Mother-Child, Lausanne University Hospital, University of Lausanne, Lausanne, Switzerland; 2 Department of Paediatrics, Hospital Neuchâtel, Neuchâtel, Switzerland; 3 Centre for Primary Care and Public Health (Unisanté), University of Lausanne, Lausanne, Switzerland; 4 Department of Neonatology, University Hospital Zurich, University of Zurich, Zurich, Switzerland; 5 Department of Neonatology, University Children’s Hospital Basel, Basel, Switzerland

**Keywords:** Bronchopulmonary dysplasia (BPD), Prediction, Respiratory distress syndrome (RDS), Risk score, Very low birth weight (VLBW) infant, Prematurity

## Abstract

Our aim was to develop and validate a predictive risk score for bronchopulmonary dysplasia (BPD), according to two clinically used definitions: 1. Need for supplementary oxygen during ≥ 28 cumulative days, BPD28, 2. Need for supplementary oxygen at 36 weeks postmenstrual age (PMA), BPD36. Logistic regression was performed in a national cohort (infants born in Switzerland with a birth weight < 1501 g and/or between 23 0/7 and 31 6/7 weeks PMA in 2009 and 2010), to identify predictors of BPD. We built the score as the sum of predicting factors, weighted according to their ORs, and analysed its discriminative properties by calculating the area under the ROC (receiver operating characteristic) curves (AUCs). This score was then applied to the Swiss national cohort from the years 2014–2015 to perform external validation. The incidence of BPD28 was 21.6% in the derivation cohort (*n* = 1488) and 25.2% in the validation cohort (*n* = 2006). The corresponding numbers for BPD36 were 11.3% and 11.1%, respectively. We identified gestational age, birth weight, antenatal corticosteroids, surfactant administration, proven infection, patent ductus arteriosus and duration of mechanical ventilation as independent predictors of BPD28. The AUCs of the BPD risk scores in the derivation cohort were 0.90 and 0.89 for the BPD28 and BPD36 definitions, respectively. The corresponding AUCs in the validation cohort were 0.92 and 0.88, respectively.

*Conclusion*: This score allows for predicting the risk of a very low birth weight infant to develop BPD early in life and may be a useful tool in clinical practice and neonatal research.

**Table:** 

**What is Known:** *• Many studies have proposed scoring systems to predict bronchopulmonary dysplasia (BPD).* *• Such a risk prediction may be important to identify high-risk patients for counselling parents, research purposes and to identify candidates for specific treatment.*	
**What is New:** *• A predictive risk score for BPD was developed and validated in a large national multicentre cohort and its performance assessed by two indices of accuracy.* *• The developed scoring system allows to predict the risk of BPD development early but also at any day of life with high validity.*

**Supplementary Information:**

The online version contains supplementary material available at 10.1007/s00431-021-04045-8.

## Introduction

Bronchopulmonary dysplasia (BPD) is a major chronic respiratory complication of preterm infants [1]. Since BPD was described for the first time more than five decades ago [2], perinatal care has seen major changes. In the 1960s, invasive mechanical ventilation was the only way to manage respiratory distress syndrome (RDS), and such exposure was shown to injure premature lungs. Different therapeutic or preventive strategies like antenatal steroids, surfactant, ventilation strategies and postnatal steroids have been studied to reduce the incidence of BPD [3, 4].

Although the administration of antenatal steroids is associated with a reduction of serious adverse outcomes related to prematurity (perinatal and neonatal death, RDS, intraventricular haemorrhage, necrotising enterocolitis), no reduction in chronic lung disease has been shown [5]. Since the 1990s, prophylactic surfactant therapy has dramatically improved survival [6]. However, the analysis of studies on the routine use of nasal continuous positive airway pressure (nCPAP) showed a decrease in ‘BPD or death’ in infants stabilised on nCPAP [7], rendering the practice of general prophylactic surfactant application obsolete.

Already 20 to 30 years ago, many research groups identified risk factors and developed scoring methods to predict BPD [8, 9]. However, those scores were rapidly outdated due to significant changes in the management of very low birth weight (VLBW) infants or due to changes in BPD definitions. More recent publications used other variables to build new predictive scoring systems [10–12]. A 2013 systematic review concluded that existing clinical predication models for BPD cannot be used in practice due to low statistical quality, and suggested that future predictive scores should be validated before implementation [13]. A new BDP imaging score performed with lung ultrasound was recently evaluated and showed promising results [14, 15]. Risk stratification of BPD might be useful for counselling families and clinical trials. More importantly, it may help to identify patients at high risk for BPD by pre-selecting them for specific treatment approaches like postnatal steroid administration, even if the optimal type, dosage and timing are still not well defined [16].

Our objective was to develop and validate a predictive BPD risk score by identifying risk factors based on a national database.

## Methods

### Design and data collection

This was a national multicentre registry study with prospective data collection for the development and validation of a predictive model for BPD. Patient data were extracted from the national database of the Swiss Neonatal Network (SwissNeoNet), which prospectively collects data from each of the nine Swiss neonatal intensive care units (NICUs) providing tertiary-level neonatal care. All admitted live-born infants with a birth weight < 1501 g and/or gestational age between 23 0/7 and 31 6/7 weeks postmenstrual age (PMA) were included. Data from patients born between January 1, 2009 and December 31, 2010 were analysed as the derivation cohort. Exclusion criteria were missing or incomplete data sets, congenital malformations or syndromes with a potential impact on the respiratory system, and death before fulfilling the BPD definition, therefore not allowing the analysis of the combined outcome ‘BPD or death’. The following variables, considered to have a potential impact on development of BPD, were extracted from the registry and patient records [17]: mode of delivery, gestational age (GA), birth weight (BW) and corresponding z-score, singleton or multiple pregnancy, antenatal steroid treatment, intubation in the delivery room, significant (requiring medical or surgical treatment but not if treated only with fluid restriction and diuretics or if treated prophylactically in the absence of symptoms) patent ductus arteriosus (PDA), proven infection (clinical evidence of infection as well as at least one relevant positive blood or cerebrospinal fluid culture), surfactant therapy, number of cumulative days of mechanical ventilation (MV) and anonymised NICUs. The outcomes predicted by the models were BPD28 and BPD36. BPD28 was defined as the need for supplementary oxygen (> 12 h per day) during ≥ 28 cumulative days between birth and 36 weeks PMA. The criterion for BPD36 was the need for supplementary oxygen at 36 weeks PMA. Data from patients born between January 1, 2014 and December 31, 2015 and included in the registry were analysed as the validation cohort, using the same exclusion criteria and extracting the same items as for the derivation cohort.

### Statistical analysis

Data analysis was performed using STATA 14 software (*StataCorp. 2015. Stata Statistical Software: Release 14. College Station, TX, USA*). Clinical characteristics were described by median and range for continuous variables, and numbers and percentages for categorical variables. The association between risk factors and BPD was examined by univariable logistic regression. To build the multivariable model, a backward deletion procedure was performed. All the variables with significant (*p* < 0.1) association with the outcome from univariable analysis were entered in the initial model. Then, variables with *p* value >0.05 were deleted one at a time. Only variables with *p* value <0.05 were retained in the final model. The BPD risk score was then built as the sum of these factors and weighted according to their ORs. Internal validation of the score was performed using the bootstrap method (repeated 1000 times), as described by Harrell et al. [18]. Multicollinearity was examined using commonly used diagnostic measures of collinearity: the Variance Inflation Factor (VIF) and tolerance. To assess the performance of the derived prognostic score, we examined two indices of accuracy: discrimination and calibration. Discrimination, i.e. the degree to which the prognostic score enables the discrimination between patients with favourable and unfavourable outcome, was assessed by calculation of the area under the receiver operating characteristic (ROC) curves (AUCs) in the derivation and validation cohorts. ROC analyses were performed at 36 weeks PMA and on day of life (DOL) 1. Calibration, i.e. the agreement between predicted and actual outcome, was assessed in the derivation and validation cohorts with the use of the Hosmer–Lemeshow goodness-of-fit test. Furthermore, sensitivity and specificity for the best cut-off value were assessed. The best threshold value of the score was chosen manually so as to simultaneously maximise the sensitivity and specificity. This procedure was performed for the two definitions of BPD.

## Results

**Fig. 1:**
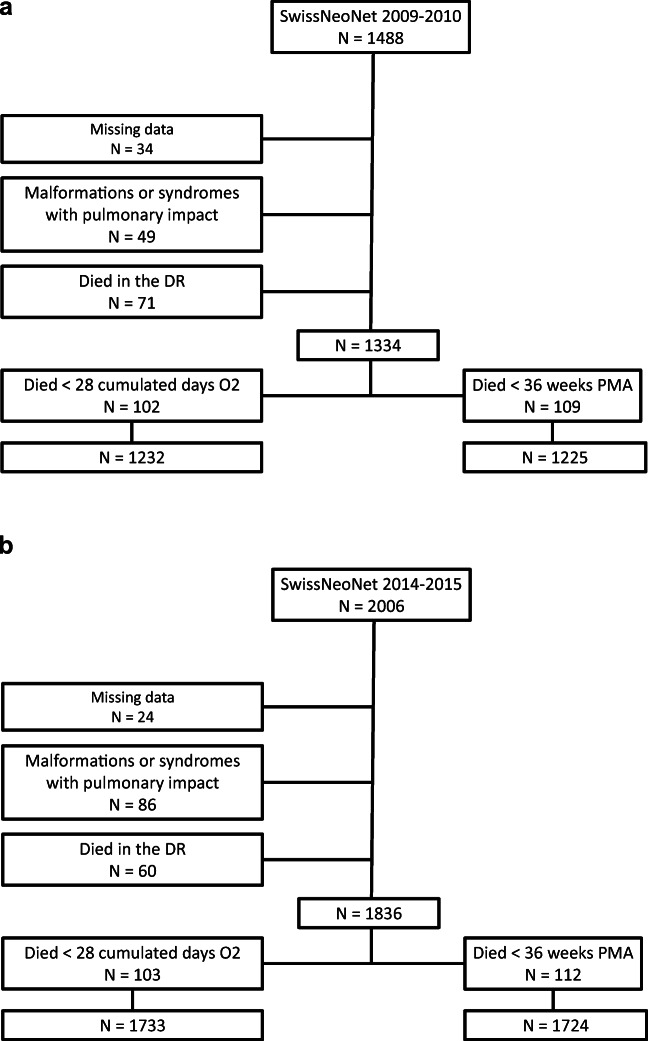
**a** Derivation cohort profile. **b** Validation cohort profile. SwissNeoNet: national database of the Swiss Neonatal Network; DR: delivery room; PMA: postmenstrual age

During the observed period for the derivation cohort (2009 and 2010), 1488 patients were born in the SwissNeoNet. Among them, 34 were excluded because of missing data and 49 because of malformations or syndromes with potential impact on the respiratory system. There were 71 deaths in the delivery room. Finally, 102 and 109 were excluded because they died before having reached the BPD28 and BPD36 criteria, respectively (Fig. 1a). The incidence of BPD28 among this population was 266/1232 (21.6%) and 138/1225 (11.3%) for BPD36.

The cohort used for validation (SwissNeoNet 2014–2015) included 2006 patients. Twenty-four were excluded due to incomplete data, 86 due to syndromes or malformations with pulmonary impact, and 60 which died in the delivery room. Of this cohort, 103 and 112 patients did not reach the age permitting a diagnosis of BPD28 or BPD36, respectively (Fig. 1b). The incidence of BPD28 was 437/1733 (25.2%) and 191/1724 (11.1%) for BPD36.

Calculated VIFs varied between 1.01 and 3.12 (no VIF >10) which indicate the absence of multicollinearity between the retained variables in the BPD28 and BPD36 BPD risk score models.

Seven variables were identified as independent predictors of BPD28 in a multivariable logistic regression analysis: GA, BW, antenatal corticosteroid treatment, surfactant administration, proven infection, PDA requiring medical or surgical treatment, and duration of MV in days. For each covariate in the model, the *β-*coefficient was directly related to the corresponding OR (Table 1).

**Table 1:** Univariate and multivariate logistic regression analysis for the BPD28 definition

Clinical characteristics	Prevalence		Univariate		Multivariate		β-coefficient
	BPD (+)	BPD (−)	BPD (+)		BPD (+)		
			OR	*P value*	OR	*P value*	
TOTAL *n* (%)	266 (21.6)	966 (78.4)					
Antenatal steroid *n* (%)	237 (92.6)	894 (89.1)	0.66	0.014	0.60	0.001	− 0.51
GA weeks median (range)	27 (23.8–31.7)	30.3 (24.3–31.9)	0.45	< 0.0001	0.67	< 0.0001	− 0.40
BW g median (range)	850 (360–1990)	1330 (460–2240)	0.66	< 0.0001	0.89	0.128	− 0.11
Surfactant therapy *n* (%)	215 (80.3)	328 (34.0)	8.2	< 0.0001	3.15	0.001	1.15
MV days median (range)	6 (0–81)	0 (0–95)	9.02	< 0.0001	2.79	< 0.0001	1.03
Proven infection *n* (%)	79 (29.7)	70 (7.3)	5.41	< 0.0001	2.3	< 0.0001	0.85
PDA *n* (%)	156 (58.7)	179 (18.5)	6.23	< 0.0001	1.90	< 0.0001	0.64

*BPD* bronchopulmonary dysplasia, *BW* birth weight, *GA* gestational age, *MV* mechanical ventilation, *PDA* patent ductus arteriosus

The BPD28 risk score derived from the *β-*coefficients was

−0.40 × GA* − 0.12 × BW* − 0.51 × antenatal steroid** + 0.64 × PDA** + 0.85 × proven infection** + 1.15 × surfactant** + 1.03 × days of MV* − 2.45 (*continuous variables were corrected and centrated as explained in Annex 1; **for categorical variables—yes = 1 and no = 0).

**Fig. 2:**
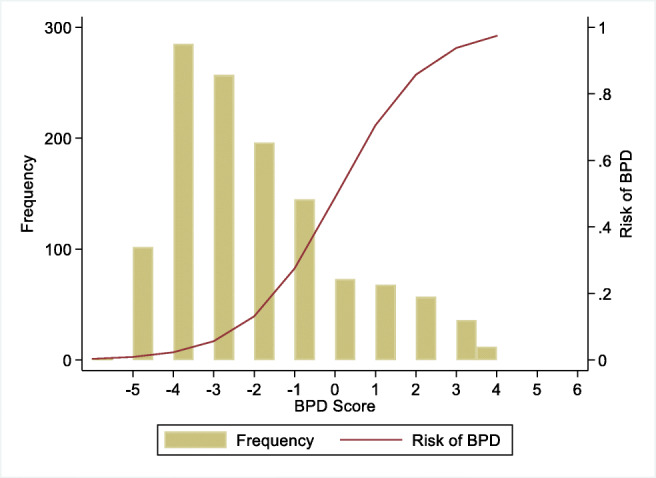
Correlation between the BPD risk score and the probability of developing BPD28. Frequency: number of patients in the validation cohort; risk of BPD: probability of developing BPD28

The association between the scores and the probability to develop BPD28 is presented in Table 2 and Fig. 2.

**Table 2:** Probability of developing BPD28 according to the BPD risk score

BPD risk score	Probability to develop BPD28
− 5	0.01
− 4	0.02
− 3	0.06
− 2	0.13
− 1	0.28
0	0.48
1	0.71
2	0.86
3	0.94
4	0.97

In the derivation cohort, maximum sensitivity (82%) and specificity (82%) were calculated at a cut-off of − 1.4 (range − 5 to + 4) at 36 weeks PMA. At DOL 1, a cut off of − 2.2 (range − 5.5 to + 0.8) for maximum sensitivity (81%) and specificity (81%) was found. The BPD risk score had an excellent discriminatory power, as shown by its AUC of 0.90 at 36 weeks PMA and 0.88 at DOL 1 and was well calibrated as confirmed by the Hosmer–Lemeshow test (Hosmer–Lemeshow *χ*^2^(28) = 24.40, Prob > *χ*^2^ = 0.66).

For the BPD36 definition, five variables were found to be statisticaly significant: BW, surfactant administration, proven infection, significant PDA and the sum of days of MV (Table 3). The model built from the *β-*coefficients was

**Table 3:** Univariate and multivariate logistic regression analysis for the BPD36 definition

Clinical characteristics	Prevalence		Univariate		Multivariate		β-coefficient
	BPD (+)	BPD (−)	BPD (+)		BPD (+)		
			OR	*P* value	OR	*P* value	
TOTAL *n* (%)	138 (11.3)	1087 (88.7)					
BW g median (range)	800 (360–1990)	1290 (450–2240)	0.68	< 0.0001	0.84	0.002	− 0.17
PDA *n* (%)	86 (62.3)	243 (22.4)	5.74	< 0.0001	1.68	0.076	0.52
Surfactant therapy *n* (%)	113 (81.9)	424 (39.0)	7.07	< 0.0001	1.87	< 0.0001	0.63
MV days median (range)	9 (0–95)	0 (0–45)	4.83	< 0.0001	2.88	< 0.0001	1.06
Proven infection *n* (%)	54 (39.1)	92 (8.5)	6.95	< 0.0001	3.62	< 0.0001	1.29

*BPD* bronchopulmonary dysplasia, *BW* birth weight, *MV* mechanical ventilation, *PDA* patent ductus arteriosus

− 0.17 × BW* + 0.52 × PDA** + 1.29 × proven infection + 0.63 × surfactant** + 1.06 × days of MV* − 3.81 (* continous variables were corrected and centrated as explained in Annex 2; **for categorical variables—yes = 1 and no = 0). The AUC of 0.89 at 36 weeks PMA and 0.84 at DOL 1 showed an excellent discriminatory power of the model, and good calibration was confirmed by the Hosmer–Lemeshow test (Hosmer–Lemeshow *χ*^2^(28) = 26.48, Prob > *χ*^2^ = 0.55). Maximum sensitivity (83%) and specificity (83%) were calculated at a cut-off of − 2.2 (range − 6 to + 2) in the derivation cohort at 36 weeks PMA. At DOL 1, a cut off of − 3.6 (range − 6 to + 2) for maximum sensitivity (77%) and specificity (77%) was found.

In the validation cohort, the performance of the score showed also an excellent discriminatory power as shown by the corresponding AUC values of 0.92 and 0.88 for BPD28 and BPD36, respectively.

As an example, for a preterm infant born at 26 3/7 weeks PMA (GA* = − 2.81), with an incomplete course of antenatal corticoids (antenatal steroid** = 1), a BW of 600 g (BW* = − 6.33), with 3 days of MV (MV* = −0.035), who received surfactant (surfactant** = 1), was treated for PDA (PDA** = 1) and had no proven infection (infection** = 0), the score would be 0.7 and the risk of developing BPD28 would be 66.3%. If the score was calculated for the same patient 1 week later after an additional 7 days of MV, the score would increase to 1.6 and therefore the probability to develop BPD would be 83%. As another example, the BPD36 risk score for this same premature infant born with 3 days of MV (MV* = 0.35), who received surfactant (surfactant** = 1), and was treated for PDA (PDA** = 1) without infection (infection** = 0) would be − 1.21 and the risk of developing BPD36 would be 22.89%. This risk would increase to 48.65% if the duration of MV increased to 10 days.

## Discussion

We developed a BPD risk score based on a large nationwide cohort and provide validation. This score allows excellent prediction of the risk of BPD early in life and at any time during hospitalisation, using relatively simple data. All predictors identified by the multivariable regression are assumed risk factors [17], except for surfactant administration and antenatal steroids. The fact that surfactant was found to be a risk rather than a protective factor in this study can be explained by the clinical reality that its administration is a marker of disease severity. To compare the differences in RDS support between the nine national NICUs, we sent them a questionnaire. The responses made it possible to consider a quite homogeneous approach in the respiratory support of VLBW infants in Switzerland. In time frame of the study in the vast majority of the Swiss units, only patients with severe RDS effectively were intubated and underwent surfactant administration, which is in line with international guidelines for the management of RDS [19]. Then, in the most recent Cochrane analysis [5], antenatal steroids did not affect the risk of BPD while a recent publication by Deshmukh and Patole [20] found that the risk was increased in extremely preterm infants below 25 WG. In the present study, antenatal steroids only were associated with reduction of BPD28 but not BPD36. Finally, lower GA and BW, proven infection, PDA and MV were associated with increased risk of BPD28 while only lower BW, proven infection, PDA and MV were for BPD36. As in any prediction model, accuracy of our BPD score was highest at the timepoint when the predicted event was supposed to occur. As the majority of our model variables are available at birth, and our sensitivity analysis revealed reliable prediction at DOL 1, we were able to demonstrate the validity of our score as of DOL 1 until 36 weeks PMA, with growing accuracy over time when more information on MV, PDA and infection become available.

In this national cohort of preterm infants < 32 weeks PMA and/or BW < 1501 g, the incidence of BPD varied widely according to the definition used. The clinical diagnosis of BPD is often used for quality control and benchmarking between NICUs [21, 22], and for the evaluation of new preventive and therapeutic strategies [23, 24]. Unfortunately, the latest consensus definition of BPD [25] is still not uniformly used in the recent literature. This is probably due to two of its shortcomings: (1) the definition requires an accurate number of hours (>12 h/day) and days of oxygen dependency, data which may be difficult to obtain in clinical practice and may not be systematically collected in patient records; and (2) in the National Institute of Child Health and Human Development (NICHD) consensus definition of BPD, nCPAP use is not considered as a possible substitute for supplemental oxygen administration and, therefore, the duration of nCPAP use is not considered in our algorithm. However, a higher mean airway pressure reduces oxygen requirement. It should be noted that nCPAP was the favoured primary mode of respiratory support in most Swiss units during the study period. According to the BPD36 definition, all infants treated with nCPAP at 36 weeks PMA would not be considered as having BPD if FiO_2_ is 0.21 (i.e. room air). If such an infant received more than 28 cumulative days of oxygen between birth and 36 weeks PMA, it would however fullfil the criteria of BPD28. Such incoherencies may in part explain the large differences between BPD incidences in the literature [26] and in our study (25.2% vs. 11.1% in the validation cohort for BPD28 and BPD36, respectively). The definition of BPD36 as used in this study may therefore be considered as a surrogate for moderate to severe forms of BPD28, according to the NICHD consensus definition.

Until now, as mentioned by Onland et al. [13], most published BPD scoring systems are only poor to moderate predictors of BPD because most of them lack validation [8, 12, 27, 28]. Furthermore, there is no published study presenting a calibration assessment of the model. In the present study, two indices of accuracy were used to assess the performance of the score. The discrimination power evaluated by the AUC was excellent and good calibration was confirmed by the Hosmer–Lemeshow test for both definitions in the derivation and validation cohorts. Otherwise, in most studies, scores assess the risk of developing BPD only at a particular moment. For example, Sinkin et al. [8] suggested a prediction at 12 h or 10 DOL, whereas Rozycki et al. [9] proposed an evaluation at 8 h of life or 14 DOL. In the study by Laughon et al. [12], prediction was made at the specific DOL 1, 3, 7, 14, 21 or 28. Compared to the Laughon score [12], our BPD score has several advantages: (1) it is based on easily assembled parameters available at any DOL until 36 weeks PMA; (2) infants of derivation and validation cohorts received comparable RDS management strategy promoting nCPAP over MV and are thus closer to typical modern day neonates; (3) the performance of the score is not only assessed by discrimination power but also by a calibration method. Promising new predicting imaging strategies using lung ultrasound are emerging [14, 15]. It would be interesting to compare this method with our model or to evaluate if the combination of both tools adds value to BPD prediction. The advantage of our score is that it is quickly achieved with existing data, and that it does not require performing lung ultrasounds, which may not be available in every NICU. Another strength of our study is its high number of patients, representing a whole population. SwissNeoNet includes > 97% of very preterm babies born in the country where patients are managed according to the best current standards with high rate of antenatal steroid treatments and preventive nCPAP. AUCs above 0.88 achieved in derivation and validation cohorts for both definitions allow an excellent prediction. Moreover, external validation brings more value to the results.

Our study presents some limitations. First, the data are extracted from a registry. Then, the scoring system was based on data coming from a country with lower BPD rate than reported internationally [29] with a quite homogeneous approach regarding respiratory support among the included tertiary centers. This model may not be applicable with the same accuracy in different settings. In addition, other variables with a potential impact on BPD such as gender or ethnicity were not included in the analysis. A further drawback is that the formula, due to its complexity, requires a calculator. To facilitate the use of the BPD risk score, we provide a web-based calculator at the following link: http://calc.chuv.ch/.

In conclusion, the presented BPD risk score allows to predict early in life and at any DOL the risk for a preterm infant to develop BPD and its potential to be moderate to severe by using seven or five easily available variables, respectively. This BPD risk score may be a useful tool in clinical practice and in neonatal research for the early identification and stratification of patients with a high risk of BPD.

### Supplementary Information

ESM 1 (PDF 296 kb)

ESM 2 (PDF 139 kb)

## Data Availability

Data cannot be made available because it would be possible to identify neonatal units for which we do not have permission.

## Annex 1

Coding: continuous variables for BPD28 definition

*Days of mechanical ventilation (MV)*:

*X* = (days of MV + 1)/100

MV1 = (*X*^−1^) − 20.99880689

MV2 = (*X*^−0.5^) − 4.582445514

MV3 = 0.0582029 × MV1 − 0.9061262 × MV2

*Days of MV** = MV3 + 0.11

*Gestational age (GA)*:

GA (days) = 7 × GA (weeks) + GA (days)

GA1 = GA (days)/7

*GA** = GA1 − 29.24

*Birth weight (BW)*:

*BW** = BW (g)/100 − 12.33

## Annex 2

Coding: continous variables for BPD36 definition

*Days of mechnical ventilation (MV)*:

*X* = (days of MV + 1)/100

MV1 = (*X*^−1^) − 21.58210007

MV2 = (*X*^−1^) × ln(*X*) + 66.29728209

MV3 = − 0.2402667 × MV1 − 0.0451578 × MV2

*Days of MV** = MV3 + 0.53

*Birth weight (BW)*:

*BW** = BW (g)/100 − 12.33

## Abbreviations

AUCs: Area under the receiver operating characteristic curves
BPD: Bronchopulmonary dysplasia
BW: Birth weight
DOL: Day of life
GA: Gestational age
MV: Mechanical ventilation
nCPAP: Nasal continuous positive airway pressure
NICHD: National Institute of Child Health and Human Development
NICUs: Neonatal intensive care units
PDA: Patent ductus arteriosus
PMA: Postmenstrual age
RDS: Respiratory distress syndrome
ROC: Receiver operating characteristic
SwissNeoNet: Swiss Neonatal Network
VIF: Variance Inflation Factor
VLBW: Very low birth weight
