# Correction to: bronchopulmonary dysplasia: a predictive scoring system for very low birth weight infants. A diagnostic accuracy study with prospective data collection

**DOI:** 10.1007/s00431-021-04105-z

**Published:** 2021-05-13

**Authors:** Ikbel El Faleh, Mohamed Faouzi, Mark Adams, Roland Gerull, Jamel Chnayna, Eric Giannoni, Matthias Roth-Kleiner, for the Swiss Neonatal Network

**Affiliations:** 1 Clinic of Neonatology, Department Women-Mother-Child, Lausanne University Hospital, University of Lausanne, Lausanne, Switzerland; 2 Department of Paediatrics, Hospital Neuchâtel, Neuchâtel, Switzerland; 3 Centre for Primary Care and Public Health (Unisanté), University of Lausanne, Lausanne, Switzerland; 4 Department of Neonatology, University Hospital Zurich, University of Zurich, Zurich, Switzerland; 5 Department of Neonatology, University Children’s Hospital Basel, Basel, Switzerland

**Correction to: European Journal of Pediatrics**

10.1007/s00431-021-04045-8

In the original published version of this article under the last paragraph of the Result section the author made changes on their presented data the corrected data are as follows [Bold text used to highlight problem area]:

## Results

As an example, for a preterm infant born at 26 3/7 weeks PMA (GA* = − 2.81), with an incomplete course of antenatal corticoids (antenatal steroid** = 1), a BW of 600 g (BW* = −6.33), with 3 days of MV (MV* = −0.035), who received surfactant (surfactant** = 1), was treated for PDA (PDA** = 1) and had no proven infection (infection** = 0), the score would be **0.7** and the risk of developing BPD28 would be **66.3%.** If the score was calculated for the same patient 1 week later after an additional 7 days of MV, the score would increase to **1.6** and therefore the probability to develop BPD would be **83%**. As another example, the BPD36 risk score for this same premature infant born with 3 days of MV (MV* = 0.35),who received surfactant (surfactant** = 1), and was treated for PDA (PDA** = 1) without infection (infection** = 0) would be − 1.21 and the risk of developing BPD36 would be 22.89%. This risk would increase to 48.65% if the duration of MV increased to 10 days.

The original article has been corrected.

